# Airborne Particulate Matter and Acute Lung Inflammation

**DOI:** 10.1289/ehp.1205860

**Published:** 2013-01-01

**Authors:** Suresh Gangamma

**Affiliations:** Department of Chemical Engineering, National Institute of Technology, Karnataka, Surathkal, India, E-mail: gangamma@gmail.com

In their article, Strak et al. (2012) connected real-world exposure to markers of acute lung function and inflammation. However, some points in the paper require further explanation. Strak et al. used fractional exhaled nitric oxide (FE_NO_) as a marker of lung inflammation. Exhaled NO is produced throughout the respiratory tract and shows significant variability in source strength across the respiratory tract (Barnes et al. 2010; Kharitonov and Barnes 2001). Factors such as particle size, hygroscopicity, composition, and concentration; lung function parameters; and environmental temperature and humidity (Varghese and Gangamma 2006, 2009), which vary across experimental locations and between participants, modify particle deposition sites in the lung. These changes in the deposition site may influence the amount of NO exhaled. In their paper, Strak et al. (2012) did not discuss how these parameters influenced their conclusions. Thus, how the linear regression model they used accounts for these influences needs to be explained.

Inflammation in the lung resulting from air pollution exposure involves various cell types, such as epithelial cells in upper airways and macrophages and recruited neutrophils in the lower respiratory tract. A significant source of exhaled NO is epithelial cells in the upper airways, which are associated with eosinophilic inflammation (Barnes et al. 2010; Kharitonov and Barnes 2001). Many components of particulate matter (PM), such as endotoxin or bacteria, induce neutrophil inflammation in the lung, but the effects of these components may not be reflected in the concentration of exhaled NO. Thus, FE_NO_ measurements as a marker of inflammation could easily be misinterpreted by attributing a particular part of the total inflammatory response within the lung to air pollution. Strak et al. (2012) did not discuss such possibilities.

In their article, Strak et al. (2012) did not provide sufficient details about the NIOX MINO monitor (Aerocrine 2010) they used to measure exhaled NO concentration. I assume that NO measurement involves flow measurement and diffusion of NO to a sensor. Temperature and humidity of exhaled air or body temperature of the subjects likely interfere with these operations. Strak et al. did not describe any of these parameters or how they may interfere with NO measurement. Moreover, the absolute values of FE_NO_ observed during the experiments are not readily available. However, in the “Discussion,” Strak et al. indicated that the observed variations between FE_NO_ measurements that are associated with particle number concentration (PNC) were most likely within the range of 5–15%. The technical specification of the instrument used for NO measurement has precision values of 5 ppb or 10% for concentrations > 30 ppb (Aerocrine 2010). Strak et al. used the difference between two sets of readings (preexposure and postexposure) as the input data for regression calculations. Thus, measurement error associated with the calculations could be much higher than that for a single set of measurements. Therefore, many of the observed differences in NO values were likely to fall within the error range of the instrument. Strak et al. should have discussed the propagation of error in the measurements or provided sufficient experimental data on the precision of the measurements. They should also have explained how the regression analysis is not biased by such instrument errors.

Strak et al. (2012) reported measurement of PNC with a condensation particle counter (CPC model 3007; TSI 2007), but their Table S2 did not report the accuracy or limit of detection of this instrument. CPC measurement depends on parameters such as ion concentration and particle composition, but because the measurements in the paper were from different environments, it is likely that these parameters varied significantly across the sites. Moreover, the CPC has a low sampling flow rate, and it is not clear whether this sampling rate is suitable for ambient measurement (aspiration efficiency in case of fluctuations in ambient wind velocity).

Overall, the article is an excellent attempt by Strak et al. (2012) to use noninvasive methods to understand the acute response of the respiratory system in response to air pollution exposure. However, a careful explanation of theory behind the experiments, experimental design, and limitations of measurement methods (if any) should have been discussed in the article.
